# Author Correction: Chromatin accessibility landscapes of skin cells in systemic sclerosis nominate dendritic cells in disease pathogenesis

**DOI:** 10.1038/s41467-020-20411-w

**Published:** 2020-12-14

**Authors:** Qian Liu, Lisa C. Zaba, Ansuman T. Satpathy, Michelle Longmire, Wen Zhang, Kun Li, Jeffrey Granja, Chuang Guo, Jun Lin, Rui Li, Karen Tolentino, Gabriela Kania, Oliver Distler, David Fiorentino, Lorinda Chung, Kun Qu, Howard Y. Chang

**Affiliations:** 1 Department of Oncology, The First Affiliated Hospital of USTC, Division of Molecular Medicine, Hefei National Laboratory for Physical Sciences at Microscale, the CAS Key Laboratory of Innate Immunity and Chronic Disease, Division of Life Sciences and Medicine, University of Science and Technology of China, Hefei, 230021 China; 2 Center for Personal Dynamic Regulomes, Stanford University School of Medicine, Stanford, CA USA; 3 Department of Dermatology, Stanford University School of Medicine, Stanford, CA 94305 USA; 4 Department of Rheumatology, University Hospital Zurich, Zurich, Switzerland; 5 Division of Rheumatology, Department of Medicine, Stanford University School of Medicine, Stanford, CA 94305 USA; 6 CAS Center for Excellence in Molecular Cell Sciences, University of Science and Technology of China, Hefei, 230027 China; 7 School of Data Sciences, University of Science and Technology of China, Hefei, 230027 China

**Keywords:** Epigenomics, Autoimmunity, Dendritic cells, Rheumatology

Correction to: *Nature Communications* 10.1038/s41467-020-19702-z, published online 17 November 2020.

The original version of this Article contained an error in the spelling of the author Lisa C. Zaba, which was incorrectly given as Lisa Zaba. This has now been corrected in both the PDF and HTML versions of the Article.

