# Supplementary material for: Chromatin accessibility landscapes of skin cells in systemic sclerosis nominate dendritic cells in disease pathogenesis
Source: Nat Commun. 2020 Nov 17;11:5843. doi: 10.1038/s41467-020-19702-z (PMC7672105; doi:10.1038/s41467-020-19702-z)
Supplement: Supplementary file 1 — Supplementary Information [file 41467_2020_19702_MOESM1_ESM.pdf]

Chromatin accessibility landscapes of cellular ecosystem in systemic sclerosis nominate dendritic cells in disease pathogenesis

Qian Liu*, Lisa Zaba*, Kun Qu†, Howard Y Chang†

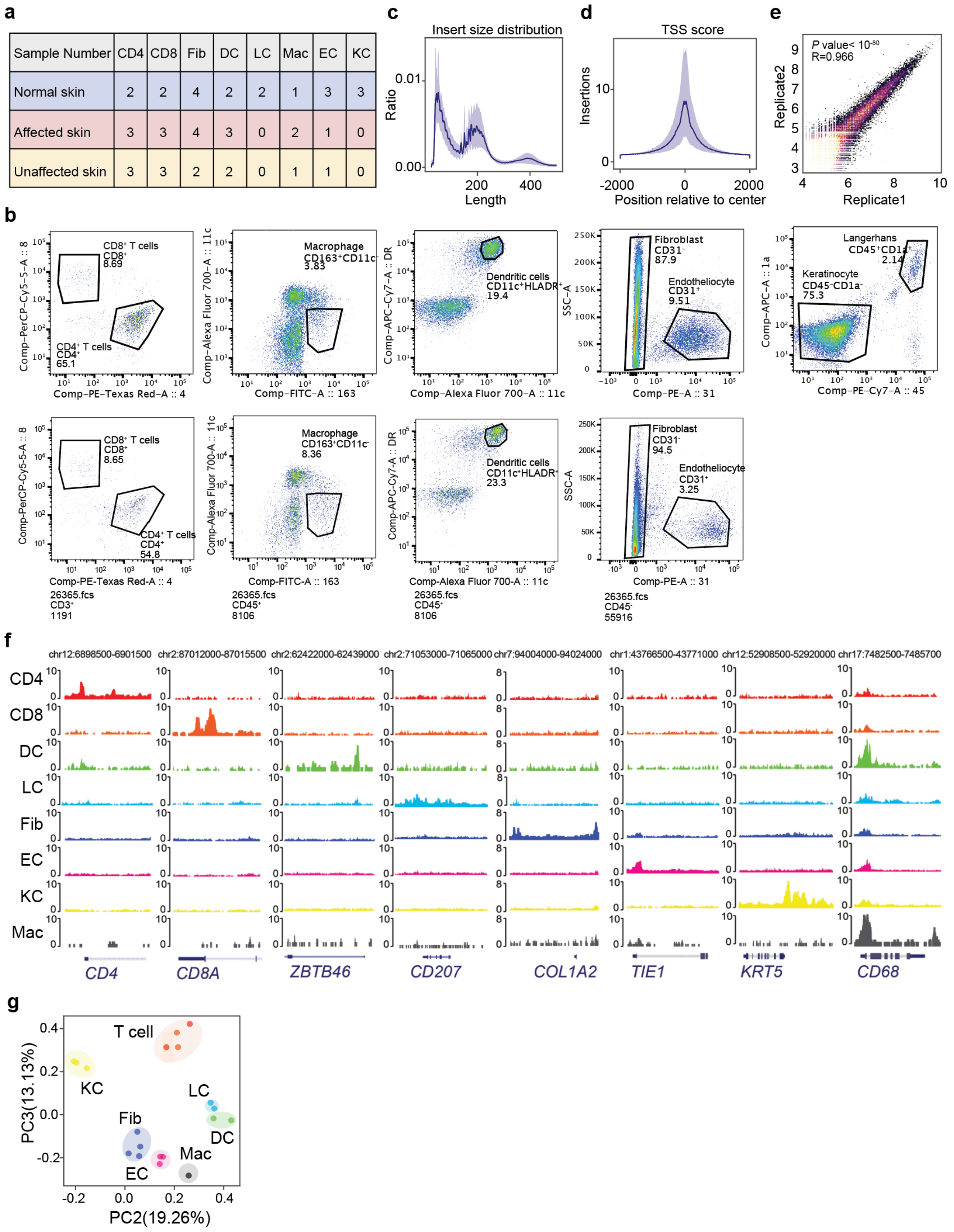

Sample Number	CD4	CD8	Fib	DC	LC	Mac	EC	KC
Normal skin	2	2	4	2	2	1	3	3
Affected skin	3	3	4	3	0	2	1	0
Unaffected skin	3	3	2	2	0	1	1	0

Supplementary Fig. 1 Quality control of ATAC-seq profiles. a. Table showing the number of biological replicates for each cell type (CD4 ($CD4^+$ T cells) and CD8 ($CD8^+$ T cells), DC (dendritic cells) and LC (Langerhans cells), Fib (fibroblasts), EC (endotheliocytes) and Mac (macrophages), and KC (keratinocytes)) in each clinical stage. **b**. Gating strategy to sort $CD8^+$ T cell ($CD45^+CD3^+CD4^-CD8^+$), $CD4^+$ Tcells ($CD45^+CD3^+CD4^+CD8^-$), macrophages ($CD45^+CD11c^-CD163^+$), dendritic cells ($CD45^+CD11c^{hi}HLA\text{-}DR^{hi}$), fibroblasts ($CD45^-$

CD31$^-$), endotheliocytes (CD45$^-$CD31$^+$), keratinocyte (CD45$^-$CD1a$^-$) and Langerhans (CD45$^+$CD1a$^+$) from normal skin (upper panels) and affected skin (lower panels). **c-d.** Plots of insert size distributions (c) and transcription start site (TSS) enrichment scores (d) of all samples from healthy donors. Light shades indicate a 95% confidence interval. **e**. Scatter plot of ATAC-seq signals for all merged peaks from a pair of biological replicates, two-tailed t-statistic *P* value and coefficient (R) of the Pearson's correlation were shown in bottom right. **f**. Normalized ATAC-seq profiles around marker genes of different cell types. **g**. Principal component analysis (PCA) of distal (distance to nearest TSS >1kb) peaks for all samples from healthy donors.

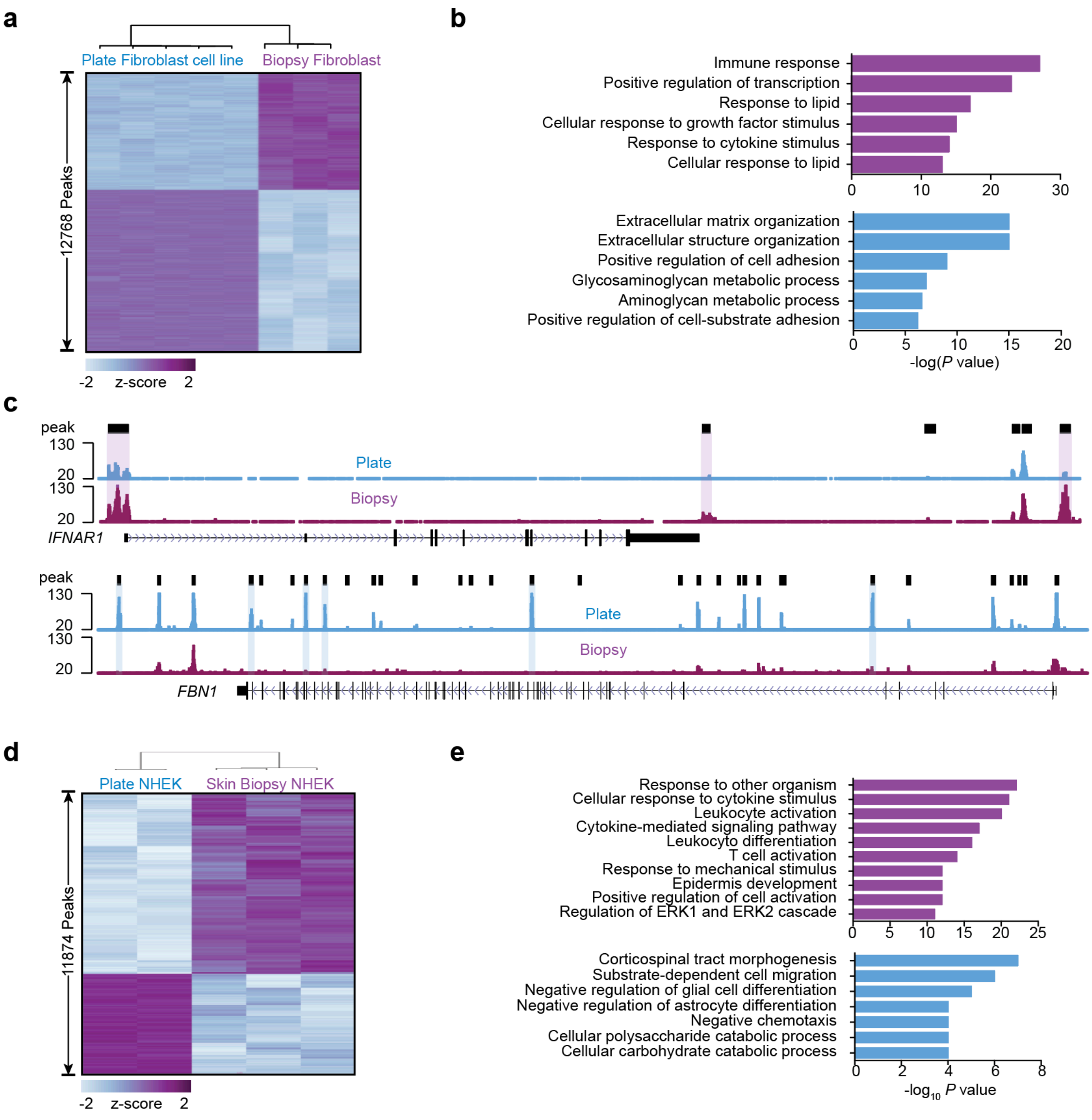

Supplementary Fig. 2 Regulome divergence of biopsy versus cultured cell. a. Heatmap of 12768 differential DNA accessible elements in fibroblasts obtained from skin biopsy and cultured cell lines in vitro. Each column is a sample; each row is an element. Samples and elements are organized by 2D unsupervised hierarchical clustering. The color scale displays relative ATAC-seq signals in z-scores as indicated. Source data are provided as a Source Data file. **b**. Top enriched gene ontologies of peaks more accessible in fibroblasts from skin biopsies (top) and *in vitro* cultured BJ cell lines (bottom), *P* values (Binom Raw *P*-value) were calculated using the binomial statistic test in GREAT. **c**. Normalized ATAC-seq signals at the *IFNAR1* and *FBN1* loci in fibroblasts from skin biopsies and cell lines cultured *in vitro*. **d**. Heatmap of 11874 differential DNA accessible elements in keratinocytes obtained from skin biopsies versus in vitro cultured cell lines. Each row is a peak, and each column is a sample. Samples and peak were organized by 2D unsupervised hierarchical clustering. Source data are provided as a Source Data file. **e**. Top enriched gene ontologies of peaks more accessible in keratinocytes from skin biopsies (top) and in vitro cultured cell lines (bottom), *P* values (Binom Raw P-value) were calculated using the binomial statistic test in GREAT.

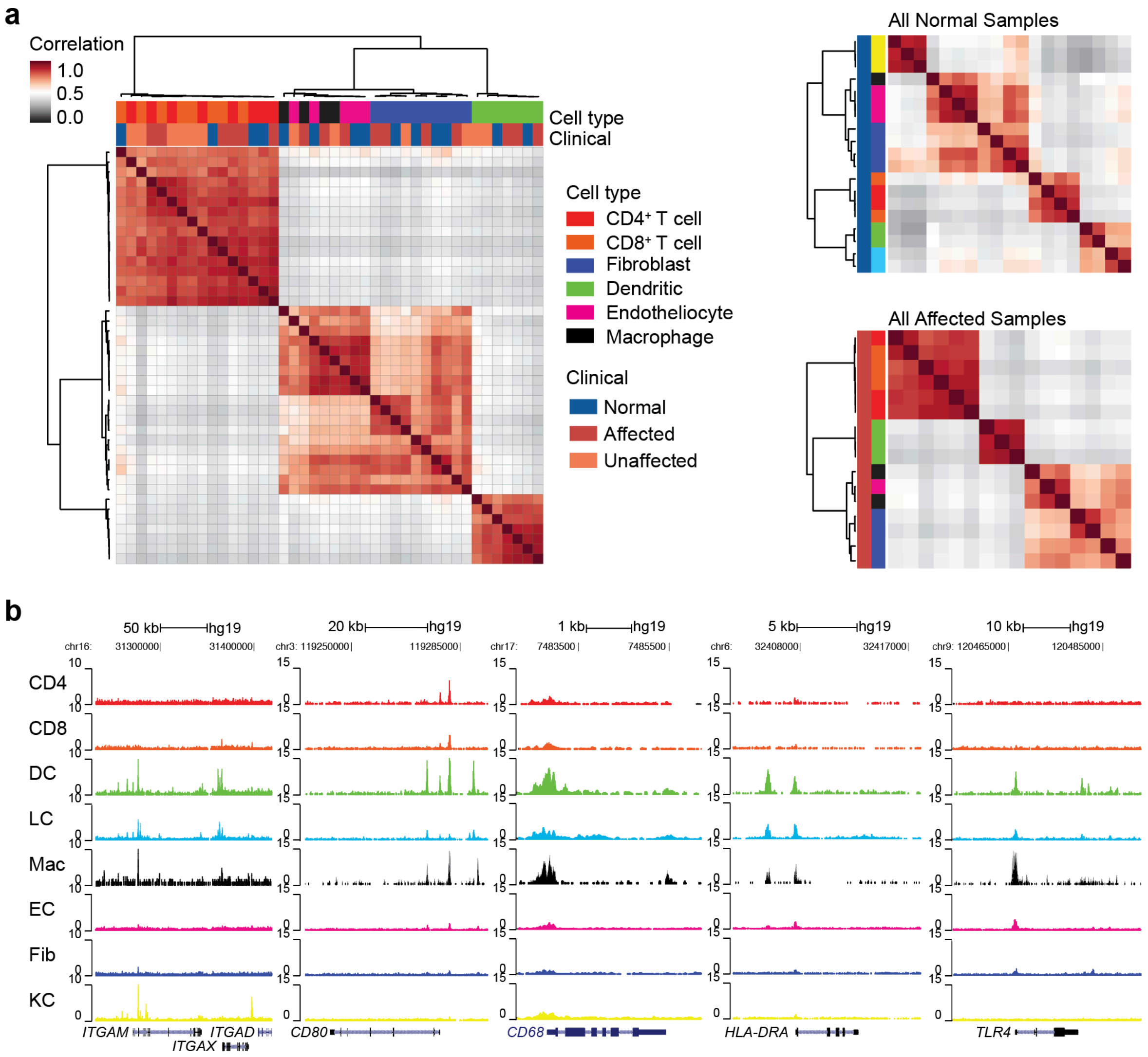

Supplementary Fig. 3 The chromatin accessible pattern of macrophage and other skin cell types. a. Unsupervised hierarchical clustering of the Pearson correlations between all the samples. ATAC-seq signals were obtained from distal elements (distance to promoter >1 kb). Left panel showing the correlation across samples of all cell types shared by healthy controls and SSc patients. Upper right and lower right panel showing the correlation across all normal and SSc affected samples respectively. Source data are provided as a Source Data file. **b**. Normalized ATAC-seq profiles around *ITGAX*, *CD80*, *CD68*, *HLA-DRA*, *TLR4*.

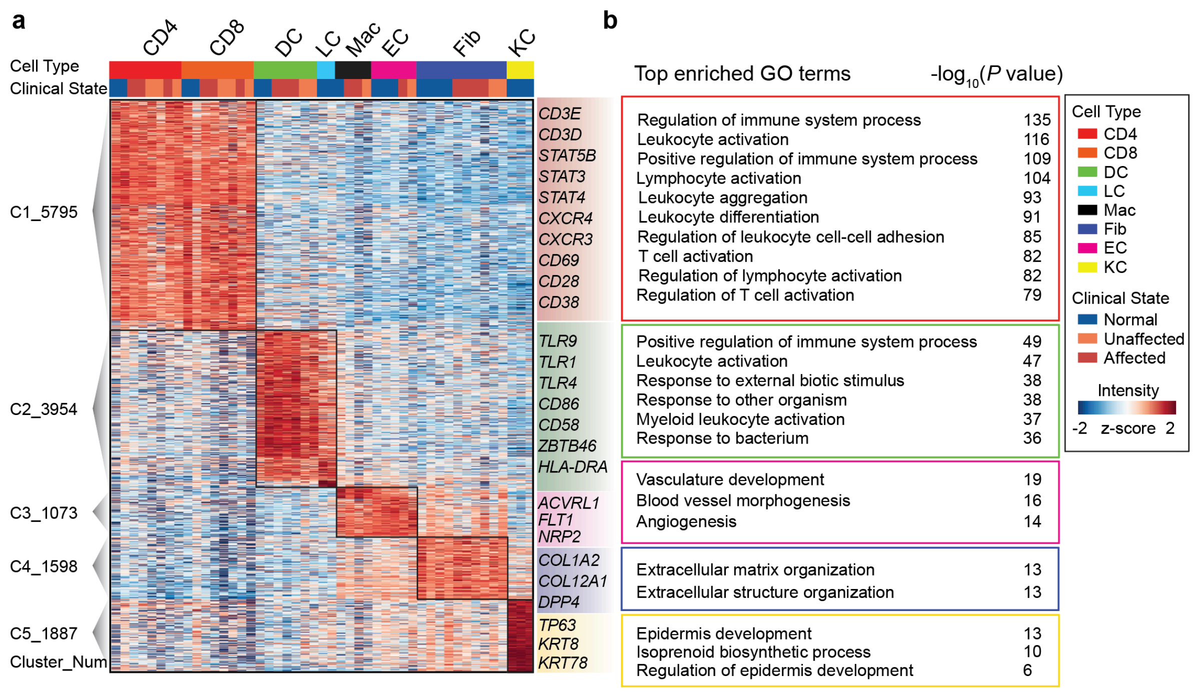

Supplementary Fig. 4 Cell type-specific chromatin accessibility in skin biopsy from both healthy donors and SSc patients. a. Heatmap of the normalized ATAC-seq intensities of cell type-specific peaks from healthy control and SSc patients. Each row is a peak, and each column is a sample, with color coded cell types (top panel). Clusters shown in the sidebar represent cell type-specific peaks of CD4 ($CD4^+$ T cells) and CD8 ($CD8^+$ T cells) (C1), DC (dendritic cells) (C2), Mac (macrophages) (C3), Fib (fibroblasts) (C4) and EC (endotheliocytes) (C5) respectively. Functional marker genes in each cluster were shown on the right. Source data are provided as a Source Data file. **b.** Top enriched GO (Gene Ontology) terms of peaks in each cluster. *P* values (Binom Raw *P* value) were calculated using the binomial statistic test in GREAT. Source data are provided as a Source Data file.

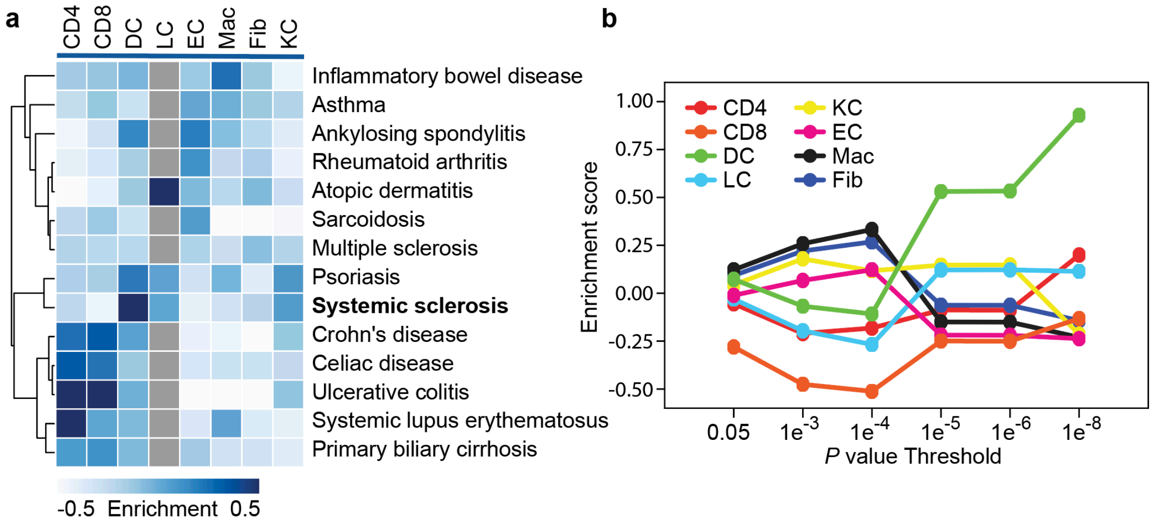

Supplementary Fig. 5 GWAS enrichments across skin cell types. a. Heatmap of the enrichment scores of manually selected immune or skin-related disease/trait-associated SNPs (single nucleotide polymorphisms) in different cell types (CD4 ($CD4^+$ T cells), CD8 ($CD8^+$ T cells), DC (dendritic cells), LC (Langerhans cells), EC (endotheliocytes), Mac (macrophages), Fib (fibroblasts) and KC (keratinocytes)) harvested from normal skin. The enrichments of non-skin-related diseases on LC were marked in gray. Source data are provided as a Source Data file. **b**. Line chart showing the enrichment score of SSc-associated SNPs under distinct cut-offs of *P* values in different cell types. Source data are provided as a Source Data file.

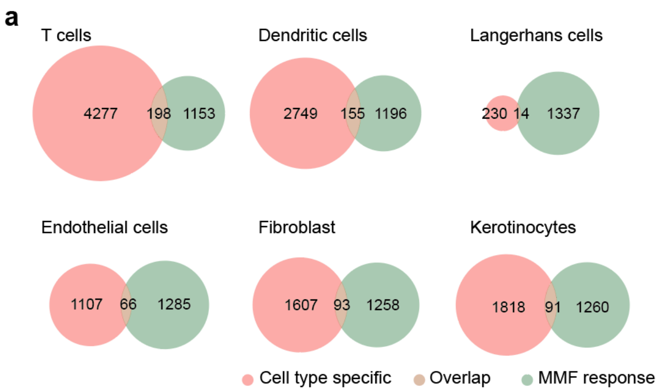

Supplementary Fig. 6 a. Venn Diagram showing the overlap of the MMF response genes and signature genes of each cell type.

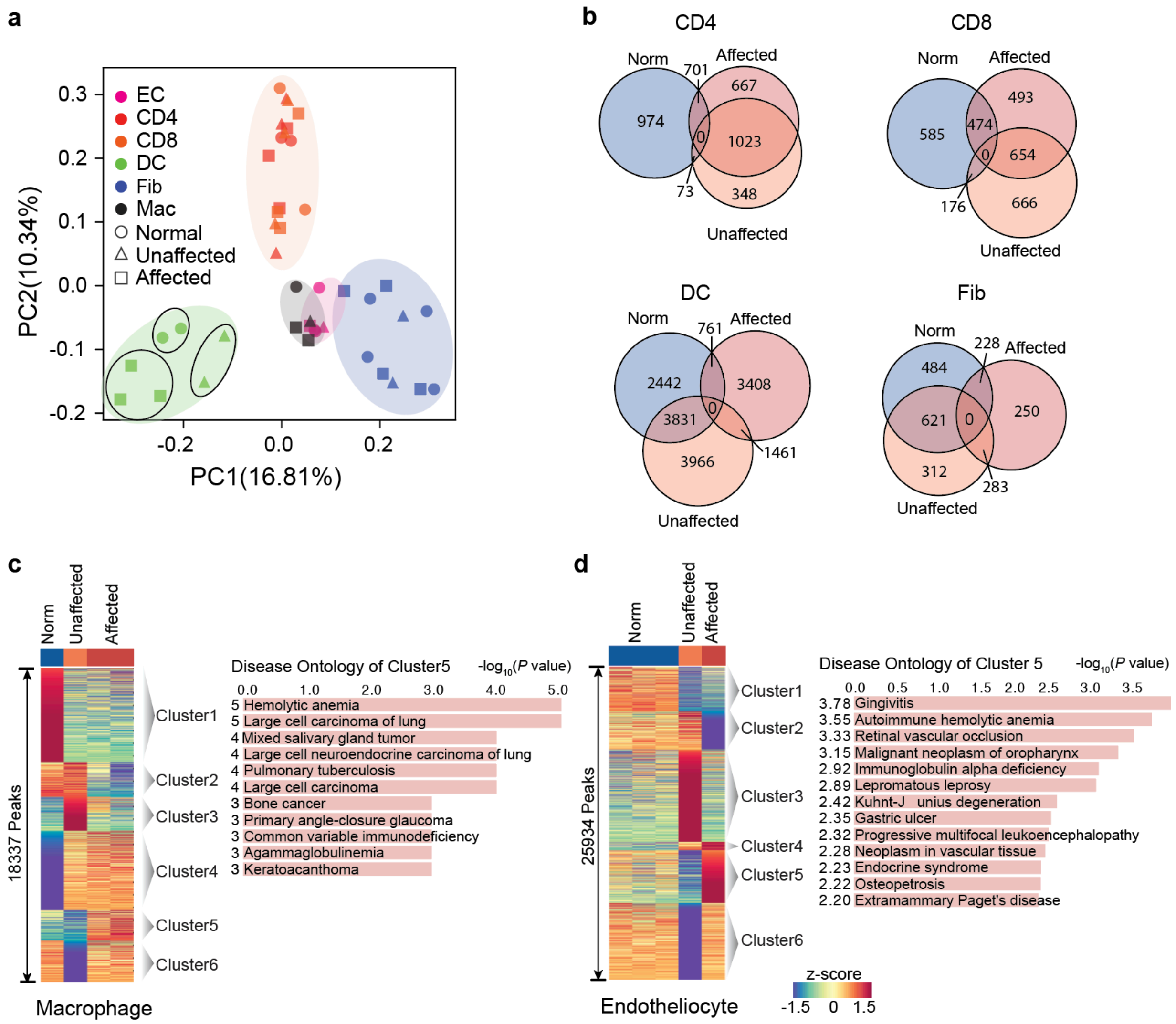

Supplementary Fig. 7 Cell type-specific regulome divergence in healthy, unaffected and affected skins. a. Principal component analysis of all ATAC-seq profiles in EC (endotheliocytes), CD4(CD4$^+$ T cells), CD8 (CD8$^+$ T cells), DC (dendritic cells) and Fib (fibroblasts). Each dot represents a sample distinguished by color (cell type) and shape (clinical state). **b**. Venn diagram showing the number of peaks in Cluster1-6 in Fig. 4e-f. Area of normal, normal-unaffected, unaffected, unaffected-affected, affected and affected-normal represent Cluster1-6 respectively. **c-d**. Heatmaps of the normalized ATAC-seq intensities (z-score) of peaks enriched in normal, unaffected and affected macrophage(c) and endotheliocyte(d). Cluster 1-6 represent the peak groups enriched in normal only, normal and unaffected, unaffected only, unaffected and affected, affected only, and normal and affected cells respectively. Each row is a peak and each column is a sample. Bar plot on the left showing the disease annotation of peaks enriched in cluster 5. *P* values (Binom Raw *P* value) were calculated using the binomial statistic test in GREAT. Source data are provided as a Source Data file.

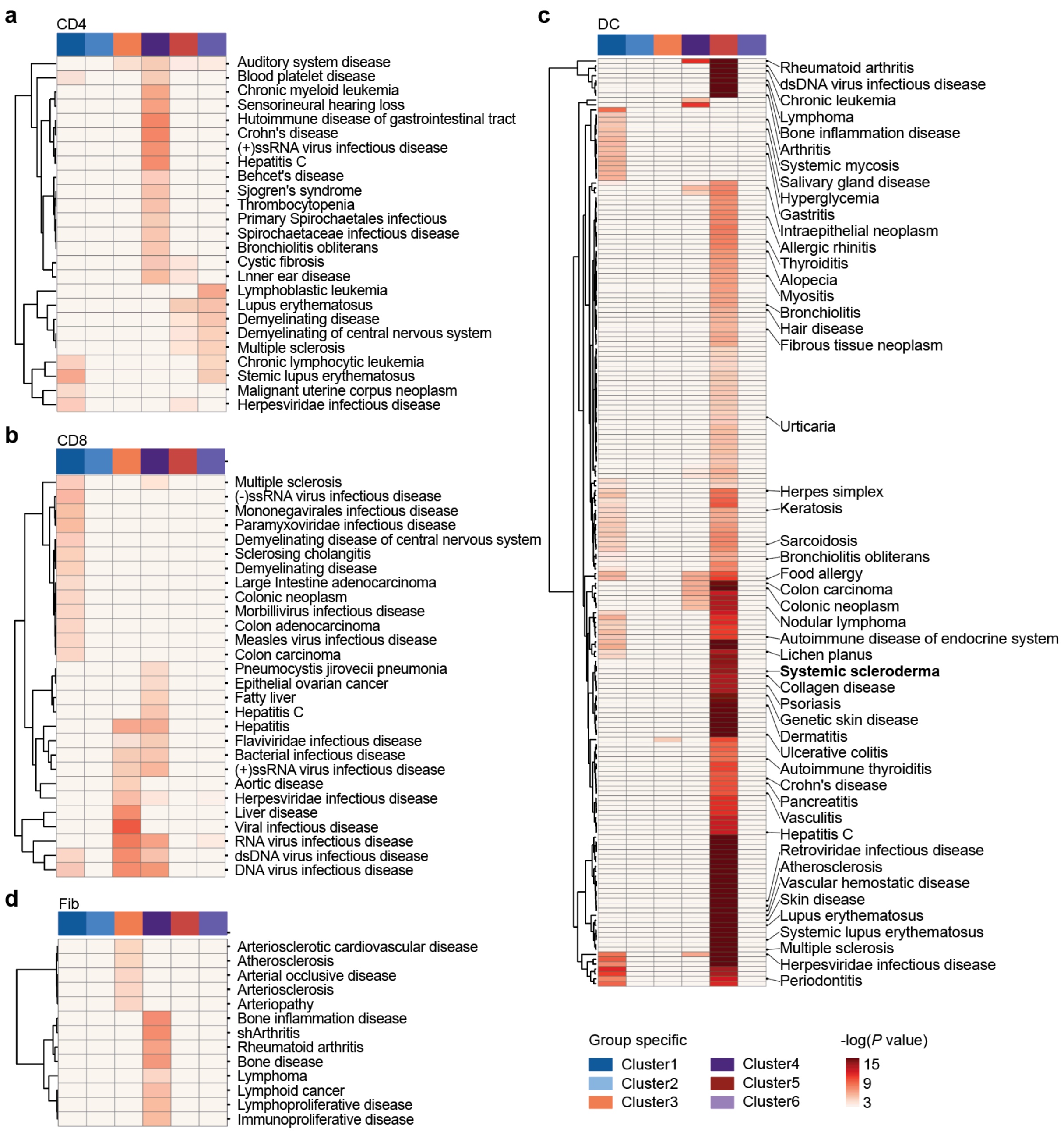

Supplementary Fig. 8 Disease Ontologies of significant differential peaks. a-d. Heatmap showing the enrichment of disease ontologies in all peaks in Cluster1-6 in Fig. 4e-f. For each cell type (CD4(CD4^{+} T cells), CD8 (CD8^{+} T cells), DC (dendritic cells) and Fib (fibroblasts)), disease ontologies with –log (*P* value)>3 were shown. *P* values (Binom Raw *P* value) were calculated using the binomial statistic test in GREAT. Source data are provided as a Source Data file.

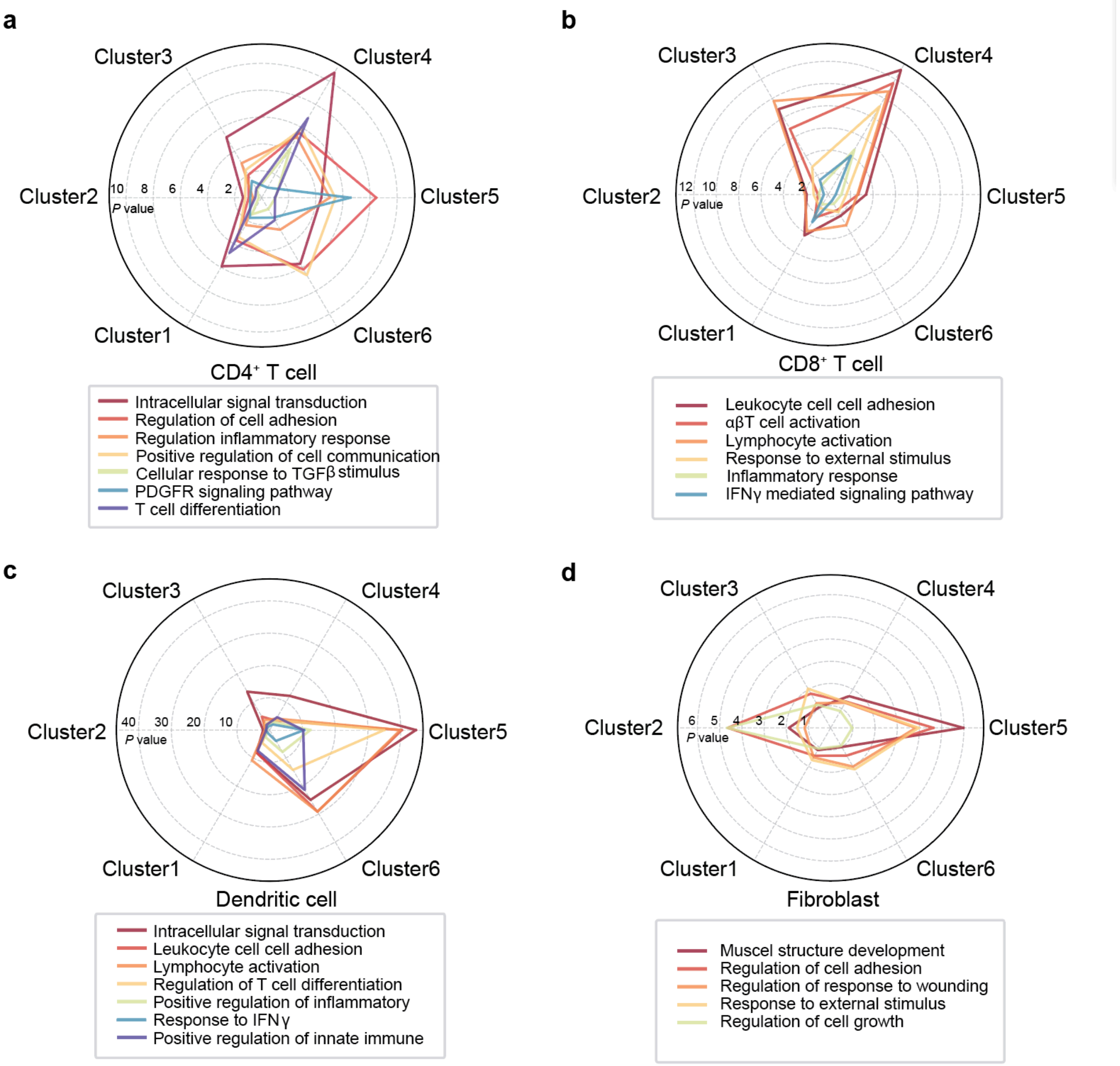

Supplementary Fig. 9 Cell type-specific regulome divergence in healthy, unaffected and affected skins. a-d. Spider plots of top enriched biological functions for each peak cluster in $CD4^+$ T cells (a), $CD8^+$ T cells (b), dendritic cells (c), and fibroblasts (d). Each angle represents a peak cluster showed in Fig. 4a-d, and the positions of the points on the radius at each angle represent the enrichment *P* values (-$\log_{10}$) of the biological functions for each peak cluster, *P* values were calculated using a hypergeometric test.

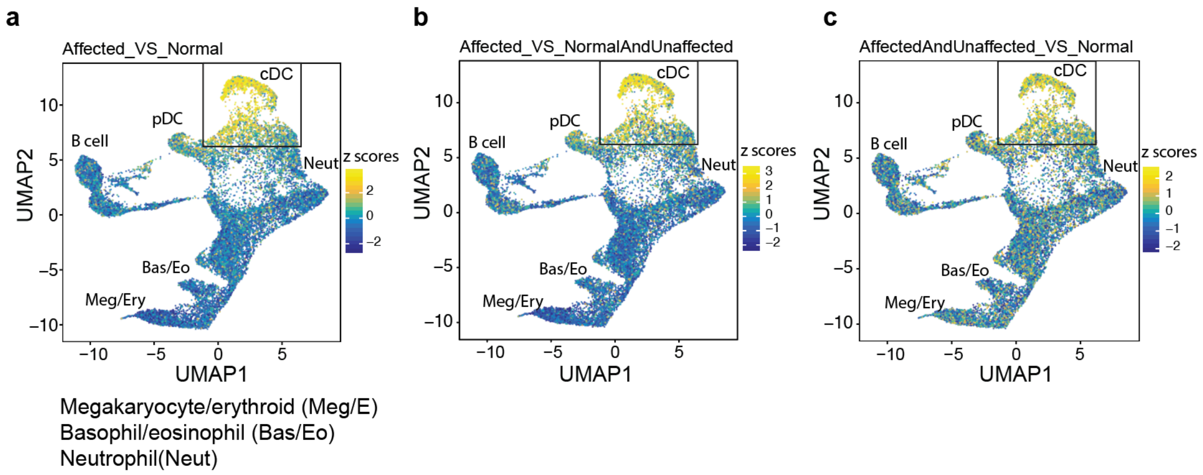

Supplementary Fig. 10 Disease signature defines cDC as the main pathogenetic DC subtype. a-c. UMAP projection of published scATAC-seq of CD34^{+} bone marrow progenitors (pDC(plasmacytoid dendritic cells), Neut (neutrophils), Bas (basophils), Eo (eosinophils), Meg (megakaryocytes), Ery (erythrocyte)) and cDC (conventional dendritic cells) colored by chromVAR deviation z-score of signature peaks more accessible in patients. Signature peaks used in (a-c) were obtained by comparing affected vs normal, affected vs normal and unaffected, affected and unaffected vs normal respectively (FDC=1).

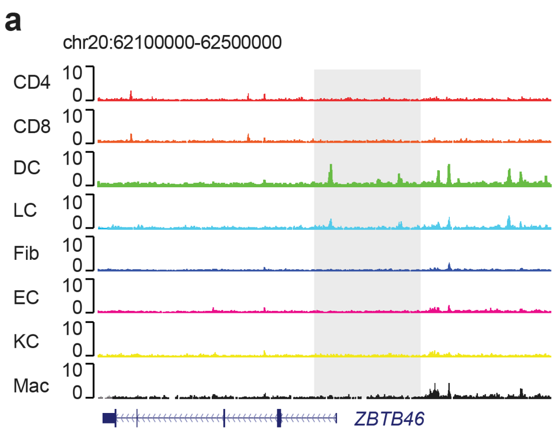

Supplementary Fig. 11 a. Normalized ATAC-seq signals at *ZBTB46* loci. in CD4 ($CD4^+$ T cells), CD8 ($CD8^+$ T cells), DC (dendritic cells), LC (Langerhans cells), Fib (fibroblasts), EC (endotheliocytes), KC (keratinocytes) and Mac (macrophages).

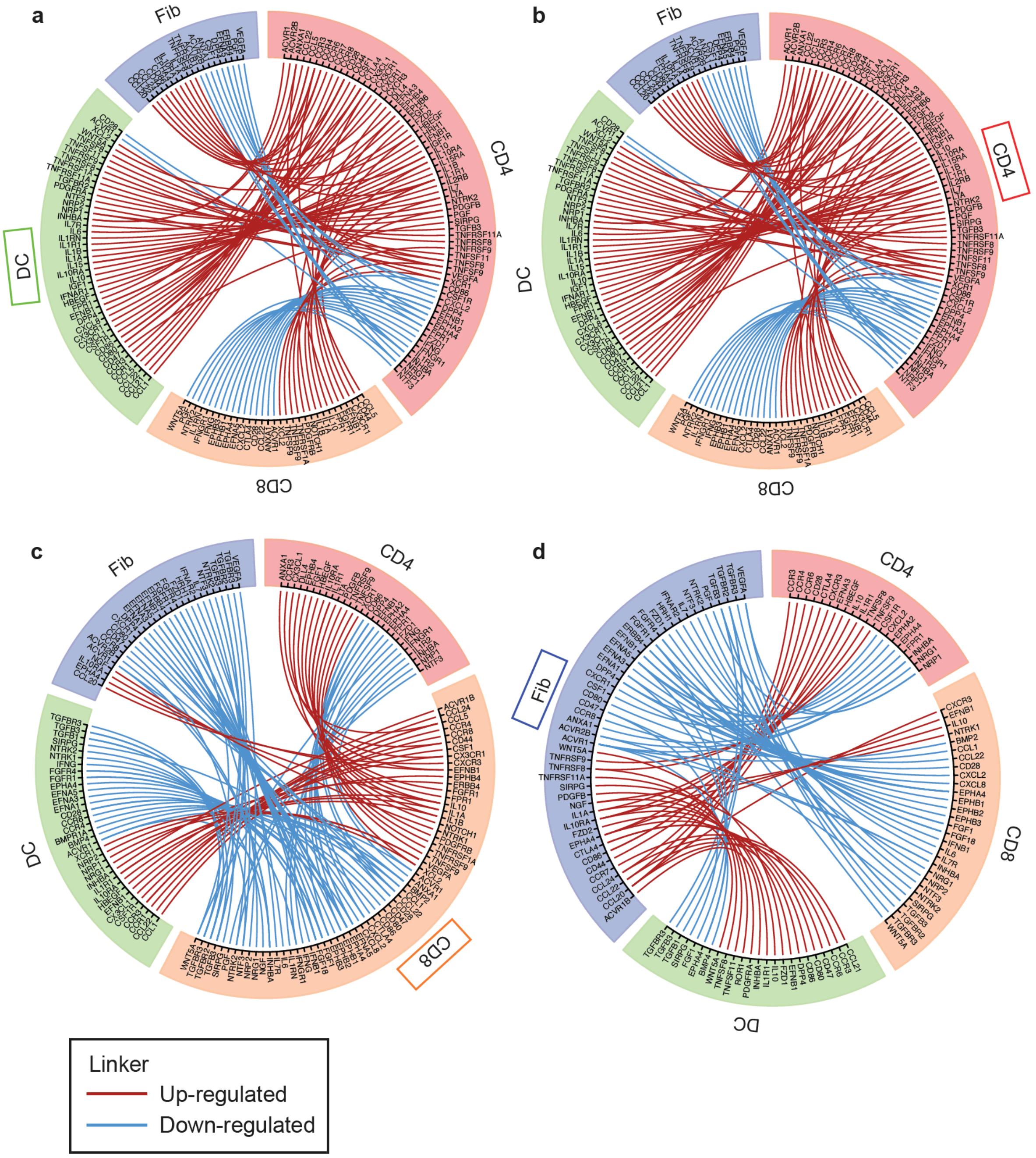

Supplementary Fig. 12 Cell-cell communications between 4 major cell types in skin. a-d. Circus plots of predicted upregulated (red) and downregulated (blue) cell-cell interactions through receptors/ligands in SSc mediated by CD4 ($CD4^+$ T cells)(a), CD8 ($CD8^+$ T cells)(b) cells, DC (dendritic cells)(c) and Fib (fibroblasts)(d). All linkers in the centre of circle in (a-d) have one end at section of DC, CD4, CD8 and Fib respectively, and are distinguished by color (up/down-regulated).
