# Supplementary material for: Chromatin accessibility landscapes of skin cells in systemic sclerosis nominate dendritic cells in disease pathogenesis
Source: Nat Commun. 2020 Nov 17;11:5843. doi: 10.1038/s41467-020-19702-z (PMC7672105; doi:10.1038/s41467-020-19702-z)
Supplement: Supplementary file 3 — Reporting Summary [file 41467_2020_19702_MOESM3_ESM.pdf]

nature research

Corresponding author(s): NCOMMS-20-01480B Howard Chang

Last updated by author(s): Oct 19, 2020

# Reporting Summary

Nature Research wishes to improve the reproducibility of the work that we publish. This form provides structure for consistency and transparency in reporting. For further information on Nature Research policies, see Authors & Referees and the Editorial Policy Checklist .

## Statistics

For all statistical analyses, confirm that the following items are present in the figure legend, table legend, main text, or Methods section.

| n/a | Confirmed | |
|---|---|---|
| ☐ | ☒ | The exact sample size ($n$) for each experimental group/condition, given as a discrete number and unit of measurement |
| ☐ | ☒ | A statement on whether measurements were taken from distinct samples or whether the same sample was measured repeatedly |
| ☐ | ☒ | The statistical test(s) used AND whether they are one- or two-sided<br>*Only common tests should be described solely by name; describe more complex techniques in the Methods section.* |
| ☐ | ☒ | A description of all covariates tested |
| ☐ | ☒ | A description of any assumptions or corrections, such as tests of normality and adjustment for multiple comparisons |
| ☐ | ☒ | A full description of the statistical parameters including central tendency (e.g. means) or other basic estimates (e.g. regression coefficient) AND variation (e.g. standard deviation) or associated estimates of uncertainty (e.g. confidence intervals) |
| ☐ | ☒ | For null hypothesis testing, the test statistic (e.g. $F$, $t$, $r$) with confidence intervals, effect sizes, degrees of freedom and $P$ value noted<br>*Give P values as exact values whenever suitable.* |
| ☒ | ☐ | For Bayesian analysis, information on the choice of priors and Markov chain Monte Carlo settings |
| ☐ | ☒ | For hierarchical and complex designs, identification of the appropriate level for tests and full reporting of outcomes |
| ☐ | ☒ | Estimates of effect sizes (e.g. Cohen's $d$, Pearson's $r$), indicating how they were calculated |

*Our web collection on statistics for biologists contains articles on many of the points above.*

## Software and code

Policy information about availability of computer code

| | |
|---|---|
| Data collection | 1) Genome:All the human data was analyzed in hg19 assembly.<br>2) Annotation: Human gene annotation were integrated from Refseq and UCSC.<br>3)Motif:All TF motifs were obtained from HOMER(Motif Analysis tools) homepage [http://homer.ucsd.edu/homer/motif/].<br>4)Receptor-ligand interaction: All interactions were downloaded from Ligand-Receptor Partners(DLRP) database [https://www.allacronyms.com/DLRP/Database_of_Ligand-Receptor_Partners] and the CellPhoneDB [https://www.cellphonedb.org/explore-sc-rna-seq]<br>5)SNP: All published disease associated-SNPs were obtained from GRASP 2.0.0.0 [https://grasp.nhlbi.nih.gov/Updates.aspx] and GWAS database [GWAS catalog:https://www.ebi.ac.uk/gwas]<br>3) Data integration:(1)ATAC-seq data collected from the human skin fibroblast BJ cell line was downloaded from Gene Expression Omnibus database, the accession number is GSE81807 [https://www.ncbi.nlm.nih.gov/geo/query/acc.cgi?acc=GSE81807] (2) Modified Rodnan skin score (mRSS) and bulk skin cell gene expression data of lesion skin biopsies of the SSc patients in treatment of mycophenolate mofetil (MMF) were obtained from Gene Expression Omnibus database, the accession number is GSE76886 [https://www.ncbi.nlm.nih.gov/geo/query/acc.cgi?acc=GSE76886]. (3)Bulk skin cell gene expression data of normal and lesion skin were obtained from Gene Expression Omnibus database the accession number are GSE130955[https://www.ncbi.nlm.nih.gov/geo/query/acc.cgi?acc=GSE130955] and GSE58095[https://www.ncbi.nlm.nih.gov/geo/query/acc.cgi?acc=GSE58095]. |
| Data analysis | ATAC-pipe_1.0.0<br>bowtie2_2.3.0<br>bedtools_2.26.0<br>homer_4.9.1<br>great_3.0.0<br>macs2_2.1.1<br><br>R packages: R version 3.5.0 |

circular_0.4.11
All data analysis notebooks are available at https://github.com/QuKunLab/SSc-ATAC-seq

For manuscripts utilizing custom algorithms or software that are central to the research but not yet described in published literature, software must be made available to editors/reviewers. We strongly encourage code deposition in a community repository (e.g. GitHub). See the Nature Research guidelines for submitting code & software for further information.

## Data

Policy information about availability of data

All manuscripts must include a data availability statement. This statement should provide the following information, where applicable:
- Accession codes, unique identifiers, or web links for publicly available datasets
- A list of figures that have associated raw data
- A description of any restrictions on data availability

No restrictions apply on data availability. The raw data and pre-processed ATAC-seq data matrix of this study have been deposited in Gene Expression Omnibus (GEO) with the primary accession code GSE99702 [https://www.ncbi.nlm.nih.gov/geo/query/acc.cgi?&acc=GSE99702].

We also used other published datasets in GEO including (1). ATAC-seq data collected from the human skin fibroblast BJ cell line, the accession number is GSE81807 [https://www.ncbi.nlm.nih.gov/geo/query/acc.cgi?acc=GSE81807]; (2). Modified Rodnan skin score (mRSS) and bulk skin cell gene expression data of lesion skin biopsies of the SSc patients in treatment of mycophenolate mofetil (MMF), the accession number is GSE76886 [https://www.ncbi.nlm.nih.gov/geo/query/acc.cgi?acc=GSE76886]; (3). Bulk skin cell gene expression data of normal and lesion skin, the accession numbers are GSE130955 [https://www.ncbi.nlm.nih.gov/geo/query/acc.cgi?acc=GSE130955] and GSE58095 [https://www.ncbi.nlm.nih.gov/geo/query/acc.cgi?acc=GSE58095].

Other data used in our paper: (1). hg19 reference genome and annotation were downloaded from UCSC[http://hgdownload.cse.ucsc.edu/goldenPath/hg19] and refseq[https://www.ncbi.nlm.nih.gov/projects/genome/guide/human]; (2). All TF motifs were obtained from HOMER(Motif Analysis tools) homepage [http://homer.ucsd.edu/homer/motif/]; (3). All interactions were downloaded from Ligand-Receptor Partners(DLRP) database [https://www.allacronyms.com/DLRP/Database_of_Ligand-Receptor_Partners] and the CellPhoneDB [https://www.cellphonedb.org/explore-sc-rna-seq]; (4). All published disease associated-SNPs were obtained from GRASP 2.0.0.0 [https://grasp.nhlbi.nih.gov/Updates.aspx] and GWAS database [GWAS catalog:https://www.ebi.ac.uk/gwas].

# Field-specific reporting

Please select the one below that is the best fit for your research. If you are not sure, read the appropriate sections before making your selection.

☒ Life sciences ☐ Behavioural & social sciences ☐ Ecological, evolutionary & environmental sciences

For a reference copy of the document with all sections, see nature.com/documents/nr-reporting-summary-flat.pdf

# Life sciences study design

All studies must disclose on these points even when the disclosure is negative.

| | |
|---|---|
| Sample size | 47 chromatin accessibility maps of 19 individuals(7 SSc patients and 12 healthy controls) from 8 cell types. Our goal was to identify the epigenomic profiles of multiple distinct primary cell types from SSc lesions, and thus each biopsy sample requires extensive cell sorting and purification to transform into multiple downstream data sets. This design is not suited for cross-sectional study of large number of patients. Our study employed a similar sized cohort as other molecular studies of human SSc tissue recently published in top journals, including Shin, J.Y. et. al. Science Translational Medicine, 2019 (n=3 SSc patients by RNA-seq of dermal fibroblast); Wohlfahrt, T. et al. Nature, 2019 (n=9 SSc patients by RNA-seq of dermal fibroblast); Maehara, T. et. al. The Journal of Clinical Investigation, 2020 (n=3 SSc patients by RNA-seq of peripheral CD4+ naïve and cytotoxicity T cell). Our study includes 28 samples from 7 SSc patients, where we produce the first genome-wide chromatin maps (in contrast to exome analysis focused on only ~2% of human genome) for multiple skin cell types rather than bulk skin cells. |
| Data exclusions | To establish a baseline normal chromatin landscape, we first harvested cells directly from fresh human skin and analyzed the genome-wide chromatin accessibility maps of 19 individuals(7 SSc patients and 12 healthy controls) from 8 cell types resident in the skin, including CD4+ and CD8+ T cells (CD4, CD8), dendritic cells (DC), Langerhans (LC), endothelial cells (EC), macrophages (Mac), fibroblasts (Fib) and keratinocytes (KC). Each ATAC-seq library was sequenced to obtain an average of more than 20 million pairedend reads, in total comprising over 380 million measurements. We used a published ATACseq pipeline,ATAC-pipe to analyze raw sequencing data and identify focal peaks of chromatin accessibility that typify active regulatory elements. After filtering and quantile normalization, we identified a total of 104,223 high quality accessible elements across these 8 skin resident cell types. |
| Replication | We did ATAC-seq data on 7 SSc patients and 12 healthy controls at healthy, SSc affected and SSc unaffected skin for 8 cell types. ATAC-seq data from healthy CD4+ T cells (CD4), CD8+ T cells(CD8), Fibroblasts(Fib), dendritic cells(DC), keratinocytes(KC), macrophage(Mac), endotheliocytes(EC) got 2,2,4,2,2,3,3 biological replicates respectively. ATAC-seq data from affected CD4, CD8, Fib, DC,Mac got 3,3,4,3,2 biological replicates respectively. ATAC-seq data from unaffected CD4, CD8, Fib, DC got 3,3,2,2 replicates respectively. We confirmed the chromatin accessibility profiles were quite similar between the biological replicates using the pearson's correlation quantification. |
| Randomization | Healthy individuals and SSc patients were randomly selected. |
| Blinding | Non-blinded study. All the researchers involved in the study were aware of the clinical status of samples (affected/unaffected/healthy samples). Experiments and data analysis were processed separately by different authors. |

# Reporting for specific materials, systems and methods

We require information from authors about some types of materials, experimental systems and methods used in many studies. Here, indicate whether each material, system or method listed is relevant to your study. If you are not sure if a list item applies to your research, read the appropriate section before selecting a response.

## Materials & experimental systems

| n/a | Involved in the study |
| --- | --- |
| ☐ | ☒ Antibodies |
| ☒ | ☐ Eukaryotic cell lines |
| ☒ | ☐ Palaeontology |
| ☒ | ☐ Animals and other organisms |
| ☐ | ☒ Human research participants |
| ☒ | ☐ Clinical data |

## Methods

| n/a | Involved in the study |
| --- | --- |
| ☒ | ☐ ChIP-seq |
| ☐ | ☒ Flow cytometry |
| ☒ | ☐ MRI-based neuroimaging |

## Antibodies

Antibodies used

| Antibody | Supplier | Catalog number | Clone | Dilution |
| --- | --- | --- | --- | --- |
| CD45 | Thermo Fisher | MHCD4530TR | HI30 | 1:50 |
| CD11c | BD Pharmingen | 561352 | B-ly6 | 1:20 |
| HLA-DR | BD Pharmingen | 335796 | L243 | 1:20 |
| CD163 | Acris | BM4041F | 5C6-FAT | 1:10 |
| CD3 BD | Pharmingen | 341101 | SK7 | 1:20 |
| CD4 BD | Pharmingen | 566914 | SK3 | 1:20 |
| CD8 BD | Pharmingen | 341049 | SK1 | 1:20 |
| CD31 BD | Pharmingen | 340297 | L133.1 | 1:20 |
| CD1a BD | Pharmingen | 559775 | HI149 | 1:20 |

Validation

CD45 Thermo Fisher MHCD4530TR HI30: The following product was used in this experiment: CD45 Monoclonal Antibody (HI30), Pacific Orange from Thermo Fisher Scientific, catalog # MHCD4530, RRID AB_10376143.
CD11c BD Pharmingen 561352 B-ly6 :Flow cytometry (Routinely Tested)
HLA-DR BD Pharmingen 335796 L243 :Flow cytometry (Routinely Tested)
CD163 Acris BM4041F 5C6-FAT:Has been described to work in FACS and Western blots.
CD3 BD Pharmingen 341101 SK7 :Flow cytometry (Routinely Tested)
CD4 BD Pharmingen 566914 SK3:Flow cytometry (Routinely Tested)
CD8 BD Pharmingen 341049 SK1:Flow cytometry (Routinely Tested)
CD31 BD Pharmingen 340297 L133.1 :Flow cytometry (Routinely Tested)
CD1a BD Pharmingen 559775 HI149 :Flow cytometry (Routinely Tested)

## Human research participants

Policy information about studies involving human research participants

Population characteristics

| Gender | age | race | Antibody | SSc subtype |
| --- | --- | --- | --- | --- |
| M | 51 | Caucasian | ANA+, RNApolIII+, Scl70-, anti-centromere - | diffuse systemic sclerosus |
| F | 52 | Caucasian | ANA+, RNAPolIII-, SCL70-, anti-centromere+ | limited systemic sclerosus |
| F | 60 | Hispanic/Latino | ANA+, RNAPolIII+, Scl70-, anti-centromere - | diffuse systemic sclerosus |
| F | 57 | Caucasian | ANA+. RNA-PolIII-, Scl70-, anti-centromere- | diffuse systemic sclerosus |
| F | 58 | Caucasian | ANA+, RNAPolIII-, Scl70+, anti-centromere- | diffuse systemic sclerosus |
| F | 40 | Caucasian | ANA+, RNAPolIII-, Scl70-, anti-centromere+ | limited systemic sclerosus |
| F | 50 | Caucasian | ANA+, RNApolIII+, Scl70-, anti-centromere - | diffuse systemic sclerosus |

Recruitment

Scleroderma patients were recruited from a clinic of new and established scleroderma patients. All patients in the clinic were asked to participate, no compensation or other benefit for participation was offered to patients other than the opportunity to participate in the advancement of medical science, and there was no apparent selection bias.

Ethics oversight

Before the skin obtaining, informed consent was obtained from each patient. Ethical approval was obtained from the Stanford Institutional Review Board (IRB) (No.27804)

Note that full information on the approval of the study protocol must also be provided in the manuscript.

# Flow Cytometry

## Plots

Confirm that:

- [x] The axis labels state the marker and fluorochrome used (e.g. CD4-FITC).
- [x] The axis scales are clearly visible. Include numbers along axes only for bottom left plot of group (a 'group' is an analysis of identical markers).
- [x] All plots are contour plots with outliers or pseudocolor plots.
- [x] A numerical value for number of cells or percentage (with statistics) is provided.

## Methodology

| | |
|---|---|
| Sample preparation | Biopsies of clinically affected and unaffected skin were obtained from arms and backs of SSc patients respectively. 5mm skin punch biopsies were digested overnight in dispase separating the epidermis and dermis, each of which were further digested separately into single cell suspension using standard protocols without ex vivo expansion. |
| Instrument | FACSAria II Flow Cytometer |
| Software | Collection: BD FACSDiva Software<br>Analysis: FlowJo version 10.6.0 |
| Cell population abundance | Post-sort purity was determined in standard fashion checking fraction of target population events/total events. Post-sort purity was greater than 90% for all populations. |
| Gating strategy | The dermis was sorted into 6 populations:<br>dendritic cells as CD45+CD11chiHLA-DRhi,<br>macrophages as CD45+CD11c-CD163+,<br>CD4+ T cells as CD45+CD3+CD4+CD8-,<br>CD8+ T cells as CD45+CD3+CD4-CD8+,<br>endotheliocytes as CD45-CD31+<br>fibroblasts as CD45-CD31-.<br>The epidermis was sorted into<br>CD45+CD1a+ LCs<br>CD45-CD1a- KCs. |

- [x] Tick this box to confirm that a figure exemplifying the gating strategy is provided in the Supplementary Information.
