# Supplementary material for: Chromatin accessibility landscapes of skin cells in systemic sclerosis nominate dendritic cells in disease pathogenesis
Source: Nat Commun. 2020 Nov 17;11:5843. doi: 10.1038/s41467-020-19702-z (PMC7672105; doi:10.1038/s41467-020-19702-z)
Supplement: Supplementary file 4 — Description of Additional Supplementary Files [file 41467_2020_19702_MOESM4_ESM.pdf]

Description of Additional Supplementary Files

Supplementary Data 1 | Cell types donated by each individual

Supplementary Data 2 | Clinical information of SSc patients

Supplementary Data 3 | Sample Informations

Supplementary Data 4 | Signature peaks and genes of each cell types in both controls and SSc patients

Supplementary Data 5 | Go Terms of Specific Signature Peaks of cell types in control samples

Supplementary Data 6 | TF enrichment in each normal samples

Supplementary Data 7 | The list of the SNPs of systemic sclerosis that were interrogated for this study

Supplementary Data 8 | GWAS enrichment score across skin cell types

Supplementary Data 9 | MMF Response Genes

Supplementary Data 10 | Signature score of each cell type in each affected skin samples

Supplementary Data 11 | Norm-Affected-Unaffected differential peaks of each cell type

Supplementary Data 12 | Disease ontologies of Norm-Affected-Unaffected differential peaks of each cell type

Supplementary Data 13| Receptor-ligand interactions up/down-regulated between CD4(CD8/DC/Fib) and other three cell types
